# Impact of lay health worker programmes on the health outcomes of mother-child pairs of HIV exposed children in Africa: A scoping review

**DOI:** 10.1371/journal.pone.0211439

**Published:** 2019-01-31

**Authors:** Kathrin Schmitz, Tariro Jayson Basera, Bonaventure Egbujie, Preethi Mistri, Nireshni Naidoo, Witness Mapanga, Jane Goudge, Majorie Mbule, Fiona Burtt, Esca Scheepers, Jude Igumbor

**Affiliations:** 1 mothers2mothers, Cape Town, South Africa; 2 School of Public Health, University of the Witwatersrand, Johannesburg, South Africa; 3 School of Public Health, University of Western Cape, Cape Town, South Africa; 4 Center for Health Policy, School of Public Health, University of the Witwatersrand, Johannesburg, South Africa; Medical Research Council, SOUTH AFRICA

## Abstract

**Background:**

Increased demand for healthcare services in countries experiencing high HIV disease burden and often coupled with a shortage of health workers, has necessitated task shifting from professional health workers to Lay Health Workers (LHWs) in order to improve healthcare delivery. Maternal and Child Health (MCH) services particularly benefit from task-shifting to LHWs or similar cadres. However, evidence on the roles and usefulness of LHWs in MCH service delivery in Sub-Saharan Africa (SSA) is not fully known.

**Objectives:**

To examine evidence of the roles and impact of lay health worker programmes focusing on Women Living with HIV (WLH) and their HIV-exposed infants (HEIs).

**Methods:**

A scoping review approach based on Arksey and O’Malley’s guiding principles was used to retrieve, review and analyse existing literature. We searched for articles published between January 2008 and July 2018 in seven (7) databases, including: MEDLINE, Embase, PsycINFO, Joanna Briggs, The Cochrane Library, EBM reviews and Web of Science. The critical constructs used for the literature search were “lay health worker”, “community health worker”, “peer mentor”, “mentor mother,” “Maternal and Child health worker”, “HIV positive mothers”, “HIV exposed infants” and PMTCT.

**Results:**

Thirty-three (33) full-text articles meeting the eligibility criteria were identified and included in the final analysis. Most (n = 13, 39.4%) of the included studies were conducted in South Africa and used a cluster RCT design (n = 13, 39.4%). The most commonly performed roles of LHWs in HIV specific MCH programmes included: community engagement and sensitisation, psychosocial support, linkage to care, encouraging women to bring their infants back for HIV testing and supporting default tracing. Community awareness on Mother to Child Transmission of HIV (MTCT), proper and consistent use of condoms, clinic attendance and timely HIV testing of HEIs, as well as retention in care for infected persons, have all improved because of LHW programmes.

**Conclusion:**

LHWs play significant roles in the management of WLH and their HEIs, improving MCH outcomes in the process. LHW interventions are beneficial in increasing access to PMTCT services and reducing MTCT of HIV, though their impact on improving adherence to ART remains scanty. Further research is needed to evaluate ART adherence in LHW interventions targeted at WLH. LHW programmes can be enhanced by increasing supportive supervision and remuneration of LHWs.

## Introduction

In countries severely affected by HIV and AIDS, shortages of health workers present a significant obstacle to scaling up quality HIV services. The increased demand for services in high HIV burdened countries on the understaffed healthcare system has resulted in task shifting to Lay Health Workers (LHWs) in order to mitigate shortage of staff and improve service delivery [1–3]. In 2008, the World Health Organization (WHO) recommended the adoption of task shifting of HIV and AIDS care where access to HIV services is constrained by a shortage of trained health workers [4].

LHWs are individuals who have not had formal tertiary or professional healthcare training and certification but have received some basic training for healthcare duties that they are required to perform within a particular intervention [4]. Given the wide geographical distribution of LHWs, there is a variety of terminology used to refer to them, including but not limited to community health workers (CHWs), village health workers (VHWs), peer counsellors and mentor mothers [5,6]. Antenatal and postnatal home visits by LHWs have been shown to improve coverage of Maternal and Child Health (MCH) services and mother-child health outcomes. A LHW programme, for instance, led to the doubling of exclusive breastfeeding among mothers living with HIV, with a 6% increase in EBF with each additional CHW visit [5].

The roles of LHWs are diverse, transcending from dealing with clients on a one-to-one basis at health facilities and in communities, interacting with the client’s family, to being involved in undertaking sensitization campaigns on HIV testing, feeding practices around HEIs, importance of male partner testing and championing implementation of health policies [4, 6]. As peer educators or counsellors, LHWs provide education on MCH, encourage early infant diagnosis (EID) of HIV, HIV counselling and testing (HCT) and antiretroviral treatment (ART) adherence [2, 3].

Although there is evidence to support the effectiveness of utilising LHWs to improve certain MCH indices such as promotion of breastfeeding, and increased uptake of childhood immunisation and cervical cancer screening, evidence remains scanty on the role of LHWs in several other specific MCH interventions such as those for HIV positive mother-HEI pairs [7]. As Sub-Saharan African (SSA) countries strengthen their systems towards elimination of mother to child transmission (elimination of mother-to-child transmission) of HIV, efforts to identify ways to improve utilisation of LHWs to deliver further MCH services that encompass all aspects of the needs of HIV positive mother and the exposed baby pairs should be intensified. Understanding how LHWs have been utilised in successful MCH programmes with a view to adopting some of the strengths of such programmes could add further impetus to drive towards elimination of mother-to-child transmission in SSA. This study is an attempt to examine and describe evidence from LHW-led interventions that have been implemented to improve health outcomes of HIV positive mothers and their HEIs.

The review aims to identify the roles played by LHWs in HIV related MCH health interventions, as well as the impact on the health outcomes of WLH and their HIV-exposed children in high HIV burden African countries.

### Commissioning agency

This scoping review presents work commissioned by mothers2mothers (m2m) that aimed to identify and synthesise research findings on current evidence of the role of LHWs in improving MCH in SSA by utilising literature from quantitative, qualitative and implementation science research. Sub-analysis of LHW’s role in HIV-specific MCH interventions was conducted to benchmark current evidence with m2m action plans.

## Materials and methods

### Study design

We conducted a scoping review in accordance with Arksey and O’Malley’s guiding framework for scoping reviews [8] and further developed by Levac and colleagues [9]. We adopted the scoping review method to summarise evidence available on LHW programmes. Scoping reviews are useful when comparing existing literature on particular interventions, where different study designs and interventions were used [8]. Arksey and O’Malley’s framework outlines an iterative and reflexive process based on six methodological stages, namely: 1) identifying the research question, 2) identifying relevant studies, 3) study selection, 4) charting the data, 5) collating, summarizing, and reporting the results, and 6) consultations[8]. We restricted our process to the first five stages.

### Data sources and search strategy

Our key research question was, “What are the roles played by LHWs in HIV related MCH interventions as well as the impact on the health outcomes of WLH and their HIV-exposed children in high HIV burden African countries?” From this, a team of three researchers identified the major constructs that included: a) Lay Health Worker b) Maternal and Child health c) HIV-exposed infants. We refined our search terms as our familiarity with literature increased as recommended by Daudt and colleagues [10] to increase broad coverage of the literature. The following databases were searched: MEDLINE, Embase, PsycINFO, Joanna Briggs, The Cochrane Library, EBM reviews and Web of Science. The search was initially conducted in March 2018 and updated in July 2018. In addition to the database searches, we hand-searched the reference lists of all relevant published studies that were returned in the search. The search strategy is presented in S1 Table and the reporting format followed the PRISMA statement as described in S1 File.

### Inclusion and exclusion criteria

Using the PICO framework, studies were included if they met the following criteria: i) HIV positive mothers alone or with their exposed baby pairs were targeted by the intervention, ii) interventions implemented in Africa for which LHWs were utilised in implementing the intervention, iii) papers with or without a comparator, iv), MCH outcomes including HIV testing, early infant diagnosis of HIV, ART adherence, viral suppression, utilisation of PMTCT services, retention in care, and knowledge of PMTCT. Additional outcomes explored include maternal depression, exclusive breastfeeding, infant growth and facility delivery rates, iv) studies published in English Language between 2008 and 2018, which coincides with the period in which WHO recommended task shifting of HIV and AIDS services, allowing lower health cadres to assume greater responsibility in HIV care delivery[8].

We included any study design, both quantitative and qualitative studies, mixed methods studies and previously published systematic reviews, to answer our research question. Systematic reviews were identified for the purpose of reviewing their included studies for potentially relevant studies. We did not conduct risk of bias as per the Joanna Briggs Institute Scoping Review Methods Manual and scoping reviews on health-related topics[11].

### Selection and data extraction

Duplicate screening of studies was done on the basis of title and abstract, and the full text. Potentially relevant studies were first identified by title by two independent reviewers. When the title proved to be inconclusive for assessing potential relevance, abstracts were read to decide whether a specific study should be included. All the identified studies were collated into EndNote for easy management of our references. Duplicates were removed from EndNote and the remaining studies were exported to Covidence software for screening. The screening process was conducted by two independent reviewers and was guided by the inclusion and exclusion criteria. Discrepancies during the screening process were resolved through discussions among the team.

We abstracted data on article characteristics: country where research was conducted; LHW programme characteristics and contextual factors, such as type of intervention, role of LHWs in intervention, frequency and intensity of engagement; barriers and facilitators to programme implementation. In addition, results of any formal assessment or evaluation of HIV specific MCH outcomes, such as MTCT rate, HIV testing, ART initiation among HIV positive pregnant and/or breastfeeding women and their infants, adherence to ART, uptake of EID of HIV and infant HIV status. Data abstraction was conducted using a standardised data extraction form that was developed *a priori* and pilot tested on a sample of five included papers. Two reviewers carried out data abstraction, and two other reviewers checked quality for consistency and accuracy.

## Results

### Search and selection of articles

**Fig 1:**
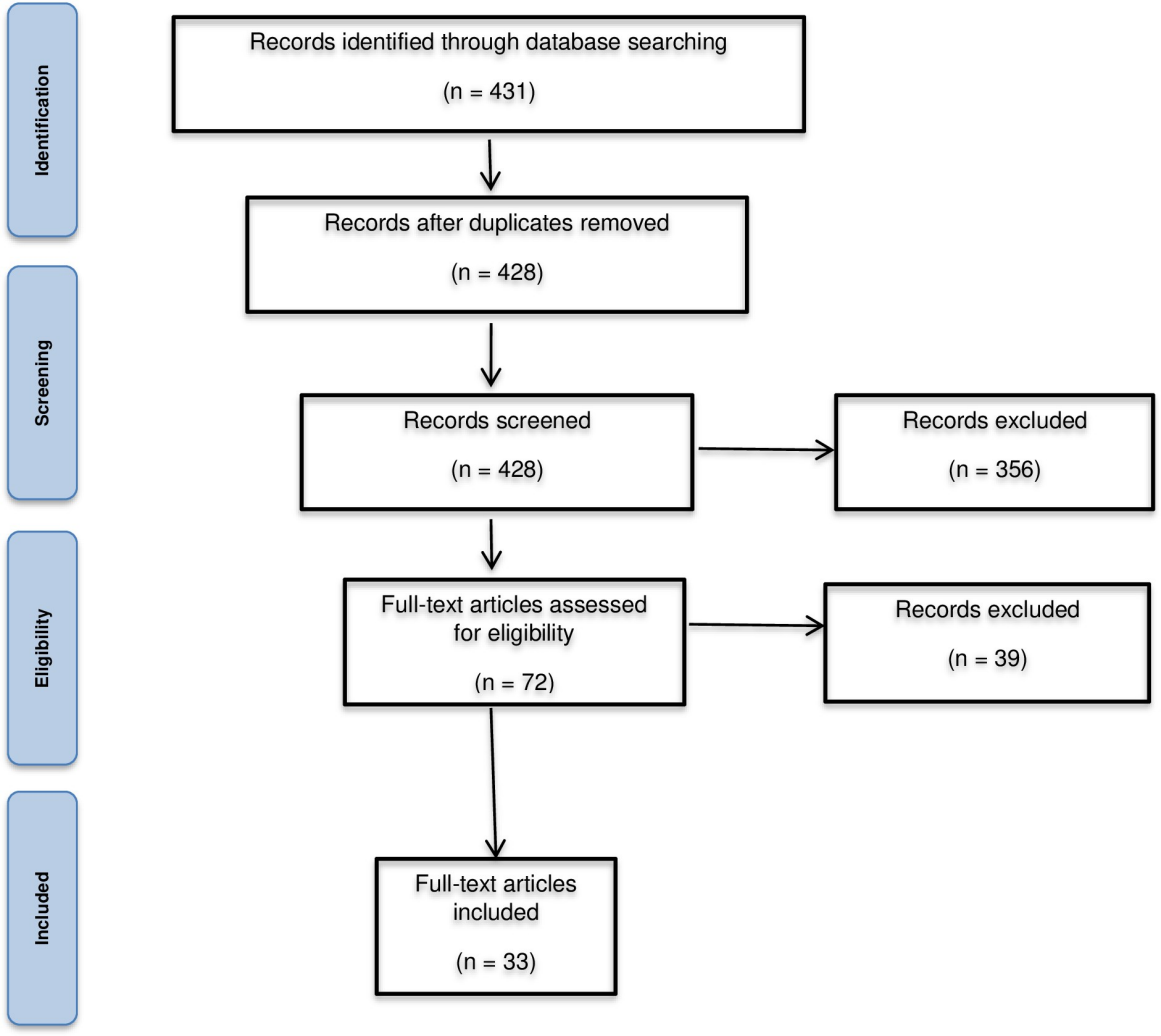
PRISMA flow diagram for the selection of studies for inclusion in the scoping review.

Our initial database search identified 431 relevant studies. After screening of titles and abstracts, 72 papers were retained. Of those, we excluded 39 articles after full-text review. Finally, 33 primary studies were identified for inclusion (Fig 1).

### Characteristics of the studies

Most studies were designed as cluster randomized controlled trials (n = 13, 39.4%), or cohort study design (n = 9, 27.3%). Other study designs in identified studies were, quasi-experimental (n = 3, 9.1%), cross sectional (n = 3, 6.1%), qualitative (n = 3, 6.1%) and RCTs (n = 3, 6.1%). Standard of care was often used as the comparative reference, in 23 of the studies. The studies originated from LHW interventions across eight countries in SSA. The highest number of studies originated from interventions in South Africa (13 studies) followed by Zimbabwe (6 studies) and Malawi with five studies, and Nigeria (3 studies), and Uganda, Tanzania, Kenya and Ethiopia each with one study. The nomenclature used to refer to LHWs was mentor mother in eight studies [6, 12–17] followed by CHW in five studies [1, 18–21] and peer mentor in three studies [22–24] and peer counsellor [25] community mobiliser/community-based cadres [2] and patient advocate[26,27].

### Roles played by LHWs

The most commonly performed roles by LHWs in MCH programmes included: health education, psychosocial support, linkage to care and defaulter tracing. In performing these roles, LHWs carry out several activities such as encouraging women to bring their babies back for HIV testing post-delivery, assisting mothers living with HIV (MLH) to access vital documentation and social grants[21, 26, 27]. A complete list of the roles of LHWs and impact of the programmes is shown in Table 1.

**Table 1:** Primary papers describing LHW interventions targeted at WLH and their exposed infants.

Authors, year & setting	Research Design	Intervention Type	Intervention implementation	Challenges to implementation	Outcome indicators	Impact of the intervention
		**Mother-to-mother / Peer Mentors**				
Richter et al, 2014 (22): South Africa	Cluster randomised control trial	Masihambisane 8 session intervention– 4 antenatal & 4 postnatal sessions for WLH attending public health clinics led by peer mentors	Antenatal to 1.5 months post-birth [intervention over 6 months]	Not all eligible women were recruited in the intervention; Some WLH did not accept the HIV test because of fear of stigma; WLH were not keen to attend ANC intervention sessions	HIV transmission-related behaviours; Infant health status; Maternal healthcare utilisation; Depression; Parenting tasks	Masihambisane had a significant effect on 3 of 16 post birth outcomes–compliance with maternal and infant ART, PMTCT tasks until 1.5 months post-delivery, more likely to request partner testing, have infants with height-for-age z-scores ≥ -2, less likely to report depressed mood and less likely to adhere to ART during pregnancy
Rotheram-Borus et al, 2014 (23): South Africa	Cluster randomised control trial	Peer mentors’ series of eight group meetings using cognitive-behavioural skills, health facilities based	From first ANC visit to 12 months post-delivery	WLH who worked or lived in the rural areas found it difficult to attend clinical services.	HIV transmission-related behaviours; Infant health status and bonding; Healthcare and health monitoring; Depression; Social support	Intervention improved 4 of 19 outcomes–one feeding method, exclusive breastfeeding for 6 months, better weight for age, larger decrease in depressed mood,
Teasdale & Besser, 2008 (17): South Africa	Cross-sectional study	Mentor mothers (HIV-positives mother who have completed PMTCT) provide comprehensive peer education and psychosocial support to pregnant women and new mothers, aimed at increasing the uptake of PMTCT services to reduce HIV MTCT, empower women and destigmatise HIV/AIDS.	Education and counselling on HIV, PMTCT, infant feeding, disclosure, safe sex, family planning, stigma. Delivered during pregnancy to 4–12 weeks post-delivery	Mentor mothers experienced suspicion and lack of trust by their clients. Mentors mothers sometimes were overwhelmed by work	PMTCT knowledge; Disclosure; Nevarapine prophylaxis; Infant feeding practice; Family planning; Referral for care	Improved ART prophylaxis for mother and infant (taken NVP and given their infants NVP for PMTCT). Increased knowledge and selected appropriate and exclusive infant feeding method. Increased disclosure of HIV status to at least one other person. Noticeable increase in mothers receiving and recalling results of CD4 count. Increased knowledge and use of contraception 4–12 weeks post-delivery. Mothers had better emotional well-being parameters.
Tomlinson et al2015(50): South Africa	Cross-sectional cohort	Mentor Mothers home visits weighing all children 6 years and under, encouraging clinical care for mothers living with HIV, following up on childhood immunizations and providing ongoing support, and the rehabilitation of underweight children	Mentor Mothers visited all homes in their neighbourhoods frequently for the first 180 days of life of each child (average of 16 visits), and every 6 months thereafter, weighing all children 6 years and under,	Some areas of the community were in accessible because reported crimes in those areas	Child growth (stunting, wasting and underweight in children)	Children living in areas where the Mentor Mothers were working were significantly less likely to be underweight and severely underweight than children living in control areas.
Phiri et al., 2017 (14): Malawi	Mixed method approach	Mentor mothers provided one-on-one support at each clinic visit, led weekly clinic-based support groups	Contact was by phone call, text message, or home visit based on the woman’s preference. Contacted women within 1 week of a missed appointment.	Mentor and expert mothers were not trained to document outcomes on clinical forms	ART uptake Retention in care	90% ART uptake compared to 86% in facility-based models. At 24 months, retention was 83% compared to 80% in facility-based models. Lower attrition rates
ENHAT-CS, 2014 (41): Ethiopia	Retrospective cohort	Mother mentors (HIV positive) followed mother-infant pairs at home and health facilities	Education and counselling individually and in Mother Support Groups on HIV, healthy behaviours, HCT for infants and partners, treatment adherence, linkage to healthcare, income generating activities, social and legal support, and tracking mothers that are lost to follow up 18 months	*Information not available	ARVs uptake; Linkage to care; HEI testing; Lost to follow-up among HEIs	Improved initiation of ART in pregnant mothers; EID through increased number of HEI tested; lower loss to follow up rates for HEIs
Shroufi et al, 2013 (16): Zimbabwe	Retrospective cohort	Mother-to-Mother mentor mother programmes for pregnant WHL	Psychosocial support, education and advice on promotion and retention in care (adherence support, counselling) 6–8 weeks	Some participants preferred receiving HIV information from formally qualified staff than mentor mothers;	PMTCT knowledge; Disclosure; Psychosocial well-being; HEI testing; PMTCT retention	Improved knowledge on PMTCT; empowered women and increased disclosure of HIV status; increased testing of HEI (EID); improved psychosocial well-being through strengthening social relationships of WLH interpersonally; improved retention in PMTCT
Futterman et al, 2010 (6): South Africa	Pilot randomised control trial	Mentor mothers Provided peer support and education through pregnancy and in the weeks following delivery. Facilitated an eight-session small group cognitive-behavioural intervention	Support through pregnancy and post-birth Knowledge of HIV & related services, ART, family planning, condom use, nutrition Disclosure strategies, managing stigma, negative emotions, domestic violence, substance abuse Infant feeding, partner testing, safe sex, adherence to postnatal infant health care & prevention activities 6 months	The programme experienced attrition of participants with time;	Adherence to PMTCT Depression	Improved knowledge of HIV and self-care–understanding the meaning and importance of viral load and CD4 test results; Significantly greater improvement in mental health; Improved social support systems; Ability to cope with HIV stigma (improved emotional states, depression scores and psychosocial coping skills); better attendance at follow-up medical visits
Besser et al, 2010 (51): South Africa	Pilot Active Client Follow-up simple intervention	Mothers-to-Mothers PMTCT peer mentors–active client follow-up; clinic and home based	Education at clinics on infant testing; reminder phone calls and home visits to promote EID and health of HEIs 16 weeks	Some of the provided phone numbers were not working; Some mothers provided wrong home addresses	EID uptake	Improved HEI testing
Sam-Agudu et al., 2017 (39): Nigeria	Prospective Paired Cohort Study	MMs link with new PMTCT clients at ANC clinics. They provide counselling and psychosocial support (including disclosure, drug adherence, and infant feeding), promote, and support early infant HIV testing. As needed, they link referred clients to higher-level care, visit, and track clients in the community to improve retention and inform facility clinical staff of ill clients needing additional care.	MMs make a first home visit within 5 days of linking with the client and visit every 2 weeks thereafter. After delivery of the infant, MMs visit their mothers within 7 days of facility delivery, or within 3 days of non-facility delivery and every 2 weeks thereafter until the infant is 12 months old. They additionally call or visit clients in the event of missed clinic appointments.	Quality of care issues at implementing PHCs. ARV and HIV test kit stock-outs, which may influence early infant testing uptake	Proportion of exposed HIV infants receiving early HIV testing by age 2 months Proportion of mothers and exposed infants retained in care at 6 months postpartum ART adherence 12-month postpartum retention MTCT rates	Exposure to structured MM support was associated with higher odds of retention than routine PS. The odds of viral suppression at 6-month postpartum were higher for MM-supported women
Sam-Agudu et al, 2017 (34): Nigeria	Prospective paired cohort study	Mentor Mothers counsel less experienced peer outcomes for optimal PMTCT outcomes	MMs make a first home visit within 5 days of linking with the client and visit every 2 weeks thereafter. After delivery of the infant, MMs visit their mothers within 7 days of facility delivery, or within 3 days of non-facility delivery and every 2 weeks thereafter until the infant is 12 months old. They additionally call or visit clients in the event of missed clinic appointments.	Implementation fidelity	Facility delivery rates	Exposure to structured MM support did not improve facility delivery
Sam-Agudu et al, 2017: Nigeria	Prospective paired cohort study	MMs link with new PMTCT clients at ANC clinics. They provide counselling and psychosocial support (including disclosure, drug adherence, and infant feeding), promote, and support early infant HIV testing. As needed, they link referred clients to higher-level care, visit, and track clients in the community to improve retention and inform facility clinical staff of ill clients needing additional care.	MMs make a first home visit within 5 days of linking with the client and visit every 2 weeks thereafter. After delivery of the infant, MMs visit their mothers within 7 days of facility delivery, or within 3 days of non-facility delivery and every 2 weeks thereafter until the infant is 12 months old. They additionally call or visit clients in the event of missed clinic appointments.	Confidentiality and fear of disclosure	Retention (clinic attendance during the first 6 month postpartum; viral suppression (viral load<20 copies/mL)	Exposure to structured MM support improved postpartum PMTCT retention and viral suppression rates
Tomlinson et al, 2017 (42): South Africa	Cluster RCT	MMs conduct home visits. They provide counselling and education on PMTCT tasks, reduced alcohol use/abuse and child growth and nutrition	MMs visit clients in their homes during pregnancy and the first 6 postpartum months for a minimum 8 times	-	Child cognitive and motor scale scores	Improved cognitive development and child growth associated with exposure to the Philani Intervention
Hosseinipour et al, 2017 (33): Malawi	Cluster RCT	MMs provide clinic-based support and community-based expert mothers provide community-based support including education and psychosocial support to all women and male partners	One-on-one support at clinics and in the community; lead weekly clinic-based support groups; lead monthly community-based support groups; Contact women within one week of a missed appointment	Failure to collect all specimens among retained women at the appropriate time points and challenges with maintaining appropriate storage conditions for dry blood spot.	Viral load suppression; Proportion of women retained at 2 years after initiating Option B+	HIV virological suppression was below the 90% desirable target
		**Peer Educators / Peer Counsellors**				
Lewycka et al, 201 (25): Malawi	Factorial cluster-randomised control trial	MaiMwana Volunteer peer counsellors, women’s groups through community mobilisation	Peer counsellors–made home visits at five time points during pregnancy and after birth to support infant feeding and care, PMTCT, family planning, care seeking	Some delays in implementation since these were volunteers; relying on self-reported statistics on breastfeeding	Maternal, perinatal, neonatal and infant mortality rate; Exclusive breastfeeding rate	Improved exclusive breastfeeding; reduction in infant and maternal mortality rate;
Sarnquist et al, 2014 (24): Zimbabwe	Quasi-experimental, prospective intervention trial	PURSE–Peers Undertaking Reproductive and Sexual Health Education Peer education on family planning for pregnant WLH 3 x 90-minute group sessions at 4 public clinics	Skills on sexual negotiation, empowerment, HIV information, PMTCT, family planning and communication skills 3 months	*Information not available	Women’s control over condom use, uptake of LARC, sexual negotiation power and ability to advocate for family planning	Improved HIV disclosure; family planning; sexual knowledge and negotiation.
Namukwaya et al, 2015 (38): Uganda	Cohort study	Peers lay persons and VHT members support women, their partners and infants through provision of health education, counselling, home visits and phone call reminders.	Pregnant women are followed through delivery and mother-infant pairs for the first 6-week postnatal visit and up to 14 weeks for EID	Some mothers declined to disclose their HIV status to the community lay persons and hence were not visited by the lay persons, but by peers instead.	Six-week attendance; EID	The peer support intervention increased six-week postnatal follow-up of HIV infected women and EID of HIV exposed infants
		**Community Health Workers**				
Kimbrough & Baker, 2014 (19): Kenya	Prospective cohort	CHWs provided health education for pregnant women, encouraged them to go to ANC visits, and urged them to deliver their babies at the health centre instead of at home or with a TBA.	HIV-positive mothers and other high-risk pregnant women were especially targeted and encouraged to deliver at the health centre	Average attrition rate of about one CHW per month. It is possible that relying on volunteers is only productive for a short time, regardless of how meaningful the work is.	Use of maternal health services (Facility based delivery	The proportion of health centre deliveries of HIV-positive women significantly increased (p = 0.04) from an average of 6.5 to 14 FBDs (115% increase).
Tomlinson et al, 2014 (21): South Africa	Cluster-randomised effectiveness trial	Good Start Community health worker intervention for maternal and newborn care and PMTCT	Antenatal and postnatal structured home visits (at least 7 visits) providing education and counselling on ANC, integrated management of childhood illnesses guided content, PMTCT, infant feeding and motivational interviewing for breastfeeding counselling. Home visits (2 during pregnancy; 1 in the first 48h after delivery; then at 3–4 days; 10–14 days; 3–4 weeks; and a final visit at 8–9 weeks)	Intervention was implemented at a time when national policy did not support EBF for HIV positive women. Low remuneration of CHWs led to shorter working hours, low motivation and sub-optimal coverage even in a situation with well-resourced supervision	Levels of HIV-free survival; Exclusive breastfeeding at 12 weeks after birth; Coverage of care; Behavioural indicators (antenatal HIV testing, a postnatal clinic visit within 7 days of life, uptake of cotrimoxazole among HIV exposed infants, and uptake of family planning) and levels of postpartum depression	The intervention almost doubled exclusive breastfeeding (EBF) at 12 weeks and showed a 6 relative increase in EBF with each additional CHW visit (With intervention having a greater effect among HIV negative women (RR 2.16 (95% CI 1.71–2.73); Improvements in knowledge of newborn danger signs, clinic visits within the first week of life, testing for HIV-exposed infants at 6 weeks and availability of cotrimoxazole in the house at 12 weeks; Increased infant weight and length for age z-scores
Nsibande et al, 2013 (1): South Africa	Community randomized trial	Good Start Saving Newborn Lives–Community health workers delivered an integrated home visit package antenatal and post-delivery	Antenatal and postnatal structured home visits (at least 7 visits) providing education and counselling on ANC, integrated management of childhood illnesses guided content, PMTCT, infant feeding; education on identifying danger signs and referrals of ill babies to health facilities. Antenatal to 12 weeks post delivery	Selection bias due to loss to follow up; recall bias due to interviews occurring 2 weeks to 18 months after the events transpired	Uptake of PMTCT and appropriate newborn care practices	High compliance with CHW referrals to health facility care for ill infants–improved linkage to care; Reduced child mortality; improve newborn health
Le Roux et al, 2013 (20): South Africa	Cluster randomised control trial	Phliani Intervention Programme (PIP) Home visits by CHWs for WLH and their infants	CHWs conducted home visits–antenatal (1–27 visits, average 6) & postnatal (1–12 visits, average 5). The antenatal messages concerned: 1) good maternal nutrition and preparing for breastfeeding; 2) regular antenatal clinic attendance and danger signs; 3) HIV testing, PMTCT tasks and partner prevention strategies; and 4) stopping alcohol use. The postnatal messages were: 1) breastfeeding and growth monitoring; 2) medical adherence (immunisations, prevention for HIV-exposed children); 3) infant bonding; and 4) securing the child grant.	The intensity of PIP programme sometimes demands CHWs to work more than their stipulated weekly hours.	Maternal nutrition & infant feeding; antenatal clinic attendance; HCT, PMTCT adherence; Infant feeding & growth; Adherence to prevention related medical care; Mother-child bonding;	The PIP programme had significant effect on 6 out of 28 outcomes: Administration of ARV prophylaxis at birth; Administration of ART to infant after birth; Practice a single feeding method first 6 months post birth Infants with a healthy height-for-age measurements; No maternal post-birth complications; Acknowledgment of infant to the family by father
Kim et al, 2012 (18): Malawi	Pilot intervention study	Tingathe-PMTCT programme Community health workers health facility and community-based tasks, followed pregnant WLH at health facilities and at home–from diagnosis at ANC to post-delivery	Ensuring all PMTCT services received, education on HIV, PMTCT care, newborn care and EID, infant feeding and nutrition, ART, managing stigma. Antenatal to testing of HEI and initiation of ART for infected infants	Some mothers refused to be followed up; Highly mobile population -some mothers moved from one area to another and were lost to follow-up	Utilisation of PMTCT, EID uptake, ART initiation	Improved initiation of pregnant mothers on ART, improved uptake of EID, early initiation of infected infants on ART
Ferrand et al, 2017 (35): Zimbabwe	RCT	CHWs had one to one session with children’s primary caregivers	Sessions were conducted for 18 months after enrolment at crucial points in a participant’s progression through HIV diagnosis, treatment initiation, and long-term care, at a location of the caregiver’s choice	Adequate ART supplies to cover 3 months were not always available	Proportion of participants who died or had a viral load of 400 copies per mL or higher at 12 months after ART initiation; Proportion who missed two or more scheduled clinic visits by 18 months post-enrolment	The community-based support program reduced risk of virological failure in HIV infected children.
Nance et al, 2017 (40): Tanzania	Cluster RCT	CHWs provided adherence counselling to pregnant and postpartum WLH and they also traced clients who defaulted	CHWs met WLH 90 days postpartum at least 1–4 times a month	Poor quality of program implementation in some facilities; Lack of CHW motivation in some sites	Retention in care between 60 and 120 days postpartum; ART initiation, timing of ART initiation and ART adherence 90 days postpartum	The CHW intervention did not have strong effects on PMTCT indicators with no significant differences in retention in care, ART initiation, or timing of ART initiation
Vogt et al, 2015 [52]: Zimbabwe	Retrospective cohort study	CHWs conducted home visits to trace defaulting patients upon request of the nurse in charge	CHWs were notified of a defaulting client residing in their area	-	Vertical HIV transmission rates 6 weeks post-partum; Retention rates during the perinatal PMTCT period; ART initiation	CHW default tracing did not reduce MTCT of HIV; Retention improved moderately during the post-natal period
		**Patient advocates**				
Jama & Tshotsho, 2013 [53]: South Africa	Qualitative study with focus group interviews using a semi-structured questionnaire	Task shifting to patient advocates already known in the community to follow up on pregnant WLH non-compliant with care	Addresses of clients were checked in maternity registers and Patient advocates visit them	Some client addresses are wrong. There were not transport for Patient advocates to use for home visits. Some WLH will run away as soon as they notice the Patient advocates’ vehicle	Tracking of non-compliant pregnant WLH to improve linkage to care	Compliance of pregnant WLH was slightly increased although there were many challenges.
Grimwood et al., 2012 (26): South Africa	Cohort study	Patient advocates provide adherence and psychosocial support for children’s caregivers; supervised the taking of medication and advised on problems that may have risen	From treatment initiation. Following the psychosocial screening visit, home visits occurred weekly for a month	Missing viral load test results	Mortality after ART initiation, patient retention, virological suppression and CD4 percentage changes on ART	Children with Pas had reduced probabilities of attrition and mortality
	Other intervention					
Patel et al, 2012 (2): Zimbabwe	Retrospective analysis of routine data	Establishment of community run early childhood development play centres for orphaned and vulnerable children (OVC) under 5 years affected by HIV, in close proximity to health centres–as an extension of PMTCT activities	Community mobilisers, village health workers, community-based carers, peer educators identified the OVC, facilitated their registration at centres, provided psychosocial support, protection, referral and linkage to health services for HIV testing and treatment. Initiated end 2009 and data evaluated September 2011	Lack of adequate resources at some of the community-run ECD	HIV testing; Initiation on ART; Community sensitisation	Improved HIV testing among children; Improved access to care and initiation on ART; Child minding capacity of carers especially on HIV care and support; Sensitisation at community level of the needs of and support for children affected by HIV
Peltzer et al, 2017 (31): South Africa	Cluster RCT	Trained LHWs facilitated counselling sessions	3 prenatal weekly 2h group sessions followed by one individual counselling session and 2 monthly individual counselling session (one prenatal, 2 postpartum)	Limited session attendance and low fidelity at several sites; Participants were not compensated for session attendance and most women found economic for transportation to the CHC for pre-and post-natal care challenging	HIV infant status, ART adherence, HIV and PMTCT knowledge	Intervention did not have any impact on HIV infant status, ART adherence, HIV and PMTCT knowledge
Mwapasa et al, 2017 (37): Malawi	Cluster RCT	Community based volunteers sent clients SMS reminders	CBVs traced mothers who miss scheduled health facility visits	Suboptimal exposure of women to the MIP service delivery model; Inadequate implementation of the study interventions by health workers; challenges in the implementation of the SMS-based tracing	Maternal and infant retention rate	SMS service delivery models were ineffective in improving maternal and infant retention

#### Health education

LHWs were reported to play a crucial role in patient education, 19 of the 33 identified articles stated that education was a key component of the intervention delivered by LHWs [6, 18, 19, 22, 24, 25, 28–30]. The range of topics covered includes HIV education, PMTCT and other post-delivery PMTCT actions, disclosure to partners and family members, safer sex practices, ART adherence and EID [23, 28, 31]. LHWs provide information and counselling with the aim of promoting breastfeeding and best infant feeding practices, family planning and immunization and elimination of MTCT [25]. LHWs also play a role in equipping mothers with skills to care for new-born babies and how to access appropriate HIV care [5, 17, 29]. Mentor mothers educate and support pregnant women and new MLH through daily educational health talks in clinic waiting rooms [16, 17] and in community support groups [6] or through one-on-one discussions during home visits [28]. The discussions focussed on initiating ART, retention in care, disclosure, coping with stigma, partner testing and disclosure, infant feeding practices, and parenting [6, 15]. LHWs educated and supported WLH and their exposed children in HCT and PMTCT attendance [15].

#### Linkage to care

LHW’s formalised links to health facilities improved their ability to influence and inform the community about where to access health services [28, 32] and encourage WLH to go to ANC visits [23], identify and channel HIV-exposed children to care [12], encourage women to bring babies back for HIV testing and to receive treatment [15]. Further, LHWs encourage WLH to deliver at health centers [23], counsel mothers on early identification of illnesses or newborn danger signs and to refer babies with illnesses [6, 21]. Mentor mothers based in clinics offer one-on-one support to women during clinic visits making them aware of the range of healthcare services available to them [33]. Community-based mentor mothers identified MLH and newly diagnosed HIV positive pregnant women and linked them to PMTCT services and ANC clinics [34]. Based on the needs of women, LHWs referred WLH to healthcare facilities for specialised professional counselling [16] and referred sick patients for higher-level care as well as informing clinical staff of patients needing additional care [34]. In Zimbabwe, CHWs referred caregivers of children living with HIV to local organisations which provided additional support services [35] and recruited them into community-based centres offering HIV services and facilitated their registration process [2].

#### Psychosocial support and defaulter tracing

For mother-infants pairs initiated into care, the LHW role is mainly centred on counselling and psychological support to promote positive living and self-efficacy in HIV management [28]. LHWs provide psychosocial support and structural support to mothers in PMTCT[36]. Mentor mothers in the Mamekhaya project in South Africa provided psychosocial support to pregnant women and new mothers to increase PMTCT uptake. The support options for mothers include supportive mentorship, coping with stigma, avoiding negative emotions, infant feeding practices, partner disclosure, safe sex practices, family planning and pre- and post-delivery care for their infants [6]. LHWs involved in community-based interventions have the potential to influence retention in HIV care by improving levels of knowledge and awareness of PMTCT for HIV and to reduce infant mortality rates by encouraging early utilisation of PMTCT services [21]. In Zimbabwe, mentor mothers provided psychosocial support, education and advice on promotion and retention in HIV care, adherence counselling and support for six to eight weeks [16]. LHWs are involved in tracking women lost to care. They send text messages to remind MLH about scheduled clinic visits [15, 37], and routine drug taking through home visits [32]. LHWs support default tracing by obtaining information about missed visits from ART providers and registers at the facility, and following up clients lost to care in the community [14, 16]. LHWs were responsible for forming adherence support groups [15, 36] and other follow-up activities including home visits centred around overcoming challenges to attending clinical appointments and collecting ART[32, 35].

### Impact of LHWs on maternal and child health outcomes

#### PMTCT knowledge, retention in care and uptake of services

Data on retention in care and utilisation of PMTCT services by mother-child pairs was reported in 14 studies. For instance, the Mamekhaya Project which provided cognitive behavioural interventions using the m2m model found that involving mentor mothers in education and support for HIV positive women increased HIV and PMTCT knowledge scores, particularly about understanding the meaning and importance of viral load, CD4 results and the efficacy of ART [6]. Similarly, a mentor mother intervention in Zimbabwe showed an increase in knowledge of PMTCT and increased retention in PMTCT continuum of care [16]. Mamekhaya was also successful in increasing by 58% the number of clinic visits by MLH compared to the control group receiving only standard of care [6].

LHW interventions in South Africa have been shown to increase ANC attendance, improve linkage to HIV care and administration of ART to infants [5, 17, 21, 29]. In South Africa, patient advocates increased retention after three years of ART in children to 91.5% compared with 86.8% among those without patient advocates. Furthermore, a 61% reduction in probability of mortality in children exposed to community-based adherence and psychosocial support compared to those without patient advocates was reported [26]. These findings were echoed by a similar mentor mother intervention in Malawi where retention was higher in facility (80%) and community-based (83%) peer support models compared to the standard of care (66%) [14], and children exposed to mentor mothers in Zimbabwe had a 99% decline in defaulter rates [16]. The PURE study in Malawi also demonstrated pre-eminence of community-based models of peer support by showing that they increased the proportion of WLH who returned after defaulting to ART (68%) compared to only 40% and 39% in facility-based models and standard of care respectively [14].

ART initiation was explored in ten studies. Follow-up visits of pregnant women by CHWs in their homes in Malawi from the time of HIV diagnosis at ANC to post-delivery resulted in improved initiation of mothers and infants on ART from baseline levels of 8.8% to 87.7% [18]. It also increased the proportion of mothers receiving CD4 results (93.6%) compared to baseline rate (22.5%) [18]. A peer-based intervention in Uganda doubled postnatal follow-up rates through home visits and phone call reminders delivered by community lay men, community lay women and village health team members [38]. Nonetheless, a CHW-based defaulter tracing programme in Zimbabwe found that while retention improved moderately during the postnatal period, retention sharply decreased after delivery, and overall the intervention had no significant impact on reduction of MTCT of HIV[39]. SMS-based methods for tracing mother-infant pairs delivered by community-based volunteers in Malawi did not impact 12-month postpartum retention of WLH and their HEIs [37]. The Protect Your Family intervention in South Africa did not affect infant and maternal adherence to PMTCT activities at 32 weeks which was attributed to the successful adoption of Option B+ (use of triple ART regardless of CD4 cell count and clinical stage of illness) in control arms [31].

Three studies reported on rate of facility delivery outcome. In Kenya, CHWs encouraged pregnant WLH to go to ANC and deliver their babies at the clinic. This led to a significant increase in the number of WLH delivering at health facilities from an average of 6.5 to 14 [19]. In Nigeria, however, structured peer support from mentor mothers did not have an impact on increasing facility delivery rates despite improving retention [40]. The time for mentor mothers to engage with clients before delivery in the Nigerian study was relatively short, thereby impacting on time for interventional support to establish significant outcomes [40].

Five studies explored the behavioural outcome of partner testing and disclosure. The Mamekhaya intervention reported no significant intervention effect on partner testing as 50% of WLH in the intervention and 45.2% of WLH in the control had a partner tested for HIV[6]. WLH attending antenatal and postnatal group sessions led by peer mentors were 16% more likely to ask their partners to test for HIV compared to women not receiving LHW support[22]. In Zimbabwe, mentor mothers facilitated partner disclosure by acting as intermediaries and providing a supportive environment for women to be confident to disclose their HIV status [16, 24]. The findings of the programme show that 98.2% of HIV-positive women enrolled in the intervention with peer support disclosed their HIV status compared to 86.2% in women without a peer-supporter [24]. A peer support intervention in Uganda increased male participation in LHW programmes for HIV related maternal and child services through psychosocial support delivered by community lay men who supported male partners of WLH and encouraged them to test for HIV, attend ANC and post-natal care and to be retained in HIV care and treatment along with their female partners [38].

#### ART adherence and viral suppression

Adherence and viral suppression were explored in 14 studies. The impact of LHWs on adherence to ART and by extension elimination of MTCT is not well elucidated. Some studies have reported benefits in favour of LHW interventions [34], while others have reported reduced adherence among intervention WLH during pregnancy compared to the standard PMTCT programme [14, 22]. The ZENITH trial in Zimbabwe found that community-based support of trained CHWs was effective in increasing children’s retention in care and adherence to treatment [35]. Consequentially, the odds of virologic failure or death were low among children receiving CHW support compared with those receiving HIV care solely at primary care facilities [35]. Similarly, exposure to structured mentor mother support was associated with a 6-fold improvement in retention and 5-fold higher odds of viral suppression at six months for supported women [34], and in Tanzania, CHW-led ART adherence counselling improved adherence in the intervention group [41]. Women attending Masihambanise meetings facilitated by peer mentors in South Africa did not only increase adherence to PMTCT behaviours, but intervention mother-infant pairs were two times likely to complete both maternal and infant ART [22].

In contrast, the PURE study in Malawi reported non-significant differences in viral suppression between HIV positive pregnant and breastfeeding mothers receiving peer support with those receiving routine PMTCT services only, HIV virological suppression was 84% which is below the UNAIDS 90% suppression desirable target in all treatment support arms [33]. These results also concur with a previous South African study showing no difference in the proportion of children achieving viral suppression between children with patient advocates (78.8%) and those without patient advocates (82.4%) [26]. The Protect Your Family intervention in South Africa did not predict infant HIV status at 6 weeks or 12 months [29]. Poor adherence to ART has been attributed to poor implementation fidelity and partial participation by WLH in LHW activities or sessions [23, 31, 34]. A CHW intervention in Shinyanga, Tanzania, improved post-partum ART adherence among MLH where the intervention was implemented with high intensity [41]. Similarly, the MoMent study in Nigeria demonstrated increased probability of retention with higher levels of attendance for mentor mother supported WLH [34].

#### Early infant diagnosis

EID was reported in seven studies. Exposure to LHW interventions increased timely presentation to clinics for EID of HIV [16], measurement and receipt of DNA PCR results reported for 80.7% of the infants in Malawi [18]. Counselling, home visiting and community sensitisation by peers increased EID from a baseline of 53.6% to 86.3% in Uganda [38]. These findings were echoed by similar mentor mother interventions in Nigeria where there was increased testing of HEIs [30, 34]. Uptake of infant HIV testing at 12 months was higher in women receiving facility-based peer support (80%) and community-based peer support (68%) compared with 60% in those receiving only standard of care in Malawi [14]. An Ethiopian study reported higher EID uptake (32% vs 15%) among infants exposed to mentor mother support compared with infants without support [42] and the Goodstart Intervention in South Africa improved EID at 6 weeks from 67% to 74% [21].

#### Exclusive breastfeeding and infant growth

Nine studies reported on the exclusive breastfeeding outcome, infant nutrition options and the implications on infant growth. LHW programs have increased rates of early and exclusive breastfeeding [17]. A volunteer peer counsellor women’s group in Malawi resulted in improved exclusive breastfeeding coupled with a 36% and 42% reduction in infant mortality rate and morbidity respectively, due to early utilisation of PMTCT [25]. The Goodstart Intervention package in South Africa increased the prevalence of exclusive breastfeeding in the intervention (29%) compared with control (15%) [21]. Peer mentor supported mothers exclusively breastfed their infants for six months, and they were more likely to use one feeding method for six months, and in turn, they had fewer underweight babies and weight for age scores [23] as well as healthy height-for-age measures [22, 29]. In South Africa, intervention children of mothers with antenatal depressed mood receiving perinatal visits from CHWs had improved cognitive development and child growth, and they were less likely to be undernourished [8, 43].

#### Family planning and safe sex

Family planning was explored in five studies. Mentor mothers facilitated the empowerment of WLH to better negotiate for condom use and to agree to family planning with partners [16]. Community integrated HIV and primary care services increased knowledge about family planning and use of effective family planning methods and safe sex [14]. HIV-positive women receiving antenatal and postnatal home visits by CHWs were 1.19 times as likely to use condoms consistently when they had sexual intercourse [20]. In Zimbabwe and Kenya, education sessions by peers aimed at increasing family planning and enhancing sex negotiation skills among pregnant WLH improved HIV disclosure, doubling use of more effective contraceptive methods from 16.7% to 36.6% [13], increased control over condom use, and dispelled myths about modern family planning methods [24].

#### Positive coping and depressive symptoms

The impact of psychosocial support on positive coping and depressive symptoms was explored in seven studies. LHW initiatives have been shown to improve mental health and ability to cope with HIV stigma, and to reduce the prevalence of depressive symptoms among WLH by helping them identify and establish social support networks [23]. Mentor mothers contributed to reducing stigma through their openness regarding their HIV status [16]. According to Futterman and colleagues, MLH receiving mentor mother support in South Africa were significantly better able to establish sources of social support and they reported a relatively high, but non-significant decline in frequency of depression [5]. Similarly, WLH attending Masihambanise peer mentor group meetings had 67% reduction in risk of depressed mood [22] as did women attending peer mentor sessions in another peer mentor program in South Africa [23]. CHWs in the Philani intervention programme were not trained to screen or treat depression, but they improved infant growth of antenatally depressed mothers by enabling them to be better carers of their infants, but without any effect on their depression [8, 43].

### Implementation challenges associated with LHW programmes

In as much as LHW programmes have shown a positive impact on HIV-specific MCH outcomes of WLH and their exposed children, their implementation faces numerous challenges. Mentor mothers found it difficult to document and complete patients’ outcome on clinical forms because they were not trained to do so [23]. In South Africa, Teasdale and Besser reported that mentor mothers experienced suspicion and lack of trust from their clients, resulting in difficulties in executing their duties [17]. Due to extra work, mentor mothers were sometimes reported to be overwhelmed by work [17]. Le Roux and colleagues [20] who indicated that the intensity of the Philani intervention programme demanded that CHWs work more than their stipulated weekly hours echoed this. In Nigeria, the implementation of the mentor mother intervention experienced challenges related to the quality of care that mentor mothers provide. In addition, the programme experienced stock-outs of HIV test kits and ART, which affected early infant testing and provision of prophylaxis [34]. The peer-counselling programme in Malawi experienced staff shortages since it relied on volunteers and this affected implementation [14]. This was also reported in Kenya, where the programme experienced attrition of at least one CHW per month due to the volunteer nature of the role [19]. Low remuneration of CHWs led to shorter working hours, low motivation and suboptimal implementation of the programme even in areas with enough supervision [32].

If LHW programmes are initiated when there is no national policy to support it, its implementation can be affected as reported by Tomlinson and colleagues where the Good Start CHW intervention was implemented at a time when national policy did not support exclusive breastfeeding for HIV-infected women in South Africa [44]. Charging a small service fee to clients seeking family planning methods in Kenya might have resulted in some clients targeted by the peer educator programme, failing to utilise the services [19].

A comparative synthesis of three implementation studies in Nigeria (MoMent), Malawi (PURE) and Zimbabwe (EPAZ) reported that common challenges to LHW programmes included involvement of male partners and concerns over confidentiality and privacy [15]. In some programmes, LHWs demanded uniforms to identify them as health workers in the community. This caused anxiety among MLH who feared being associated with HIV because other community members could easily associate the LHW home visit with an HIV positive household member [32]. Hence to protect client’s confidentiality, LHWs did not wear uniforms [32, 39]. The degree of fidelity with which the LHW intervention was implemented had an impact on the effectiveness of the intervention in South Africa [31] and Tanzania [41] where attendance by WLH to peer sessions was low. In Malawi, about 42% of health visits by mothers occurred on scheduled dates and SMS reminders for missed appointments were only sent 43% of the time [33, 37]. High attrition rates were reported in the Mamekhaya intervention with 44% attrition [6], while a LHW programme in Malawi reported attrition rates of 16.8% [18].

## Discussion

The scoping review aimed to examine the roles and impact of lay health worker programmes targeting MCH outcomes of WLH and their HIV exposed children. We found that LHWs play crucial roles in the education of mothers, linkage to care, providing psychosocial support to MLH and their HEIs, tracing defaulters, promoting exclusive breastfeeding and presentation of mothers with theirs babies to the clinic for EID of HIV. As highlighted in a previous systematic review investigating the roles of CHWs in HIV care in SSA, LHWs were reported to enhance the reach, uptake and quality of HIV services, as well as the dignity, quality of life and retention in care of people living with HIV [45].

The majority of the studies we found were conducted in South Africa. HIV and AIDS research is arguably a dynamic area of research in South Africa given the burden of HIV and AIDS in the country [31], more developed research infrastructure and more funding towards research overall.

The findings show that delegating of specific tasks to cadres of LHWs can increase access to HIV services and can improve the quality of care for HIV. The roles of LHWs have expanded to accommodate the growing needs of WLH and their HEIs, and providing a holistic coverage of care including clinical, educational and psychosocial support to prevent attrition and to keep mother-child pairs in HIV care. Well-functioning and sustainable service delivery heavily relies on support and training, resources at LHW’s disposal and the quality of implementation.

We found that uptake of PMTCT services and psychosocial support were the main target of the identified interventions, followed by interventions to increase ART initiation and EID. Importantly, WLH require knowledge on PMTCT, how to care for their exposed infants and how to live healthy [7, 17, 20, 21, 46, 47]. This may be attributed to countries in SSA putting greater emphasis on elimination of mother-to-child transmission of HIV. The existing evidence base for LHW interventions to increase disclosure is limited and shows variable results. Further research is needed to determine if current LHW approaches to increasing disclosure are effective or whether new approaches should be considered [9]. Stronger evidence and research are needed to determine the long-term effects of LHW interventions in improving retention throughout the PMTCT steps [48].

Other clinical conditions such as maternal depression and infections were subject to study to a lesser degree in our review. We suggest that future studies should better reflect the mental wellbeing and coping efficacy of HIV positive mothers, as the prevalence of maternal depression especially among postpartum women can exceed 35% [43].

Many studies described LHW interventions but most lacked clear information about details of their tasks or the methods used to interact with mother-child pairs, and frequency of contact with clients. In addition, a few explained why the standard of care intervention alone was inadequate. To enhance our understanding and eventually replicate the interventions in other settings, we recommend clarity regarding intervention adequacy and more detailed descriptions of the development of the intervention including fidelity, dosage and challenges encountered [49]. For instance, fidelity analysis of a LHW programme in South Africa reported that intervention facilities provided 58–88% of the intervention elements. However, following additional training and supervision of low fidelity staff, 75–96% of intervention content was delivered[29].

Our findings highlight that while LHWs encouraged adherence to ART, there was an insignificant impact on most LHW programmes with suboptimal adherence levels reported [26, 33, 37]. Literature indicates that adherence to ART and adherence to PMTCT programmes remain a challenge, and this may diminish the gains made in elimination of mother-to-child transmission of HIV [26, 27]. The reasons for non-adherence has been presented as non-disclosure of HIV status, stigma, and poor understanding of HIV, ART and PMTCT [48, 50, 51]. In some instances, even with the implementation of LHW interventions in combination with standard care, these obstacles remain unchanged [45]. More qualitative research is needed to evaluate ART adherence interventions and understand the perceptions and experiences of WLH on LHW programme [51].

There is paucity of data on the role of social and cultural context on the uptake of interventions within African communities. Moreover, some researchers argue that using a multifaceted approach that considers contextual settings, leadership involvement and shared learning fora will more likely yield better results. This gives credence to the argument that a “no one size fits all” approach is better suited to closing the implementation gap for priority prevention programs such as PMTCT of HIV [9]. LHW programmes have successfully recognised that HIV is multifaceted and can be managed through numerous approaches including delivering healthcare into the community [48].

Our review has its strengths and limitations. The extent of our search was broad, as we aimed to give a holistic view of the studies in the field. Our scoping review is however limited as we did not look into grey and unpublished literature. In addition, we did not screen for quality of studies, and thus studies included vary widely regarding methods and sample sizes. Also, the exclusion of non-English publications in our search could have omitted studies published in journals in non-English speaking countries. The majority of studies did not exclusively examine LHW interventions targeted at mothers living with HIV and their HEIs. Finally, the success of LHW programmes is often dependent on implementation fidelity, the scope of training provided to LHWs, and adequacy of resources. We were not able to differentiate between projects on the basis of these implementation issues.

## Conclusion

The most commonly reported roles of LHWs include health education, psychosocial support, linkage to care and defaulter tracing. Findings of this scoping review show that delegation of tasks to cadres of LHWs can increase access to care among WLH and their baby pairs, retention in care, exclusive breastfeeding, presentation of HEIs at clinics for EID for HIV, use of effective family planning methods and negotiating for safe sex. There is, however, no effect on adherence to ART, and mixed findings for impact on viral suppression, male partner testing and increasing facility delivery rates. Program quality and fidelity can be enhanced by motivating LHWs through provision of adequate remuneration, integration of LHW programs into the public health system and supportive supervision to sustain the success of interventions.

## Supporting information

S1 File PRISMA 2009 checklist. (DOC)

S1 Table Search strategy. (DOCX)
