# Supplementary material for: Impact of lay health worker programmes on the health outcomes of mother-child pairs of HIV exposed children in Africa: A scoping review
Source: PLoS One. 2019 Jan 31;14(1):e0211439. doi: 10.1371/journal.pone.0211439 (PMC6355001; doi:10.1371/journal.pone.0211439)
Supplement: S1 Table — (DOCX) [file pone.0211439.s002.docx]

**Search Strategy**

| **Search** | **Search terms** |
| --- | --- |
| #5 | #1 AND #2 AND #3 |
| #4 | Systematic review OR scoping review OR comprehensive review OR literature review OR critical review OR integrative review OR rapid review OR review of evidence |
| #3 | community health worker OR volunteer health worker OR lay health worker OR lay health advisor  OR lay health educator OR village health worker OR village health volunteer OR lady health  worker OR community health volunteer OR community health agent OR community health promotion OR community health promoter OR community health aide OR health assistant worker OR home based care  OR home community based care OR community health agent OR health surveillance assistant OR community  care giver OR community caregiver OR accredited social health activists OR accredited social health activist OR asha OR mitanin  OR family health team OR family health program OR integrated community case management OR LHW OR CHW OR VHW OR ASHA OR traditional birth attendant OR TBA OR HAS OR lay counsellor OR peer counsellor OR health mentor OR peer mentor OR mother mentor OR Health Extension Worker |
| #2 | HIV OR AIDS OR Seropositive HIV OR human immunodeficiency virus |
| #1 | Women OR female OR maternal OR pregnant OR neonates OR newborn OR children OR child OR infant |
